# Neonatal Resuscitation Practices and Associated Factors Among Nurses in Tanzanian Delivery Suites: A Cross-Sectional Study

**DOI:** 10.24248/eahrj.v9i1.831

**Published:** 2025-09-30

**Authors:** Salehe Mrutu, Edwin Lugazia, Amina Omari, Atala Jongo, Hassani Msanga

**Affiliations:** a Department of Anaesthesiology, Campus College of Medicine, School of Clinical Medicine, Muhimbili University of Health and Allied Sciences, Ilala-Dar Es Salaam.

## Abstract

**Background:**

Neonatal deaths contribute significantly to under-five mortality, with most deaths seen in sub-Saharan Africa (SSA). Birth asphyxia is the leading cause of these deaths, but it is preventable with effective resuscitation. In SSA, including Tanzania, inadequate neonatal resuscitation practice by health care providers has been identified as a significant contributor to neonatal mortality.

**Aim of the study:**

The study aimed to assess the current practice of neonatal resuscitation and the associated factors among nurses located in the delivery suites.

**Methodology:**

A hospital-based cross-sectional study was done at Muhimbili National Hospital. It involved the direct observation of 138 cases of neonatal resuscitation(NR) by 49 nurses. Proportions were used to assess practice. The chi-square and Fisher's Exact Tests were used to determine associations between provider characteristics and practice. Logistic regression models were used to identify independent predictors of practice.

**Results:**

Overall, 52% of neonatal resuscitation cases met standard guidelines. Adequate drying was performed in 82.6% of cases, but inappropriate stimulation occurred in 16.7%, and wet towels were discarded in only 38.4%. Initial assessment was correct in 49.6%, and airway opening in 73.3%. A good mask seal was achieved in 93%, yet the first rescue breath was correctly given in 32.1% and the second course in 27.2%. One newborn required chest compressions, but they were incorrectly done. Documentation post-resuscitation was done in only 2.9% of cases. Formal training (cOR 0.181; 95% CI, 0.038 to 0.871; *P*=.033), in-house training (*P*=.049), work experience (cOR 0.368; 95% CI, 0.161 to 0.839; *P*=.017), and knowledge of neonatal resuscitation (cOR 0.392; 95% CI, 0.197 to 0.781; *P*=.008 were associated with neonatal resuscitation practice. Working experience of 3 to 5 years (AOR .352; 95% CI, 0.148 to 0.836; *P*=.018) was found to be an independent predictor of practice.

**Conclusion and recommendation:**

Neonatal resuscitation is still a challenge among providers working in delivery suites. Working experience is an independent predictor of practice, but other factors, such as knowledge level, training, and ongoing in-house training, are equally important. Regular training, integration of neonatal resuscitation into continuous professional development, and embedding resuscitation performance in quality improvement frameworks are recommended.

## BACKGROUND

The World Health Organisation (WHO) reported global deaths of newborns of approximately 2.4 million in 2019. The report shows that 47% of all deaths among under-fives occurred in the newborn period, with about one-third dying at day one and about a quarter dying within the first week of life.^1^ This mortality is projected to increase to 52% by 2030 if the strategic intervention is not kept in place.^2^ The majority of neonatal deaths were reported in low and middle-income countries, including sub-Saharan Africa (SSA), and account for three-quarters of total neonatal deaths.^3,4^ The majority of these early neonatal deaths are due to birth asphyxia (BA). It is reported that around four million neonates experience birth asphyxia during delivery, and one million die annually because of birth asphyxia.^5,6^

In Tanzania, the newborn mortality rate is reported to be 21 deaths per 1,000 live births, and birth asphyxia accounts for the majority of these deaths,^7,8^ which can be averted by effective neonatal resuscitation.^9,10^

A small but significant number of newborns fail to initiate and sustain breathing at birth, which is known as birth asphyxia (BA). Without effective resuscitation, these newborns are likely to die.^11^ Neonatal resuscitation encompasses a set of skill interventions after birth to help the newborn establish breathing and circulation and to prevent morbidity and mortality caused by a lack of oxygen to the organ system.^12,13,14^ The interventions required include keeping the baby warm, assisted ventilation, and chest compression in <1% of cases.^15^ Hence, all personnel in the delivery rooms should be adequately trained in all skills of neonatal resuscitation and ready for neonatal resuscitation.^16^

Despite the overall decrease in the under-five mortality rate, the neonatal mortality rate is still a challenge.^17,18^ Neonatal resuscitation (NR) skills are of crucial importance towards achieving the Sustainable Development Goal (SDG) of reduction of neonatal mortality to 12 per 1,000 live births by 2030.^19^

Nurses are often the primary birth attendants in Tanzania's delivery suites. Understanding the current neonatal resuscitation practice and associated factors will provide a road map to device interventions that will improve newborn care and reduce preventable neonatal deaths in Tanzania.

The study aimed to assess the neonatal resuscitation practice among nurses located in the delivery suites at Muhimbili National Hospital(MNH) in relation to the standard guidelines (timely drying, stimulation, and maintaining warmth, maintaining open airway, providing assisted ventilation in the golden minute when and chest compression when needed) and identify the factors associated with the practice of neonatal resuscitation from September to December 2021.

## METHODS

### Study Design

This research employed a quantitative analytical hospital-based cross-sectional design, and it was conducted between September and December 2021.

### Study Area

The study was conducted at Muhimbili National Hospital (MNH), which is located in West Upanga, Ilala District in Dar-es-salaam. The Muhimbili National Hospital serves as the National Hospital and a teaching hospital offering a wide range of super-specialised services.

### Study Population

**FIGURE 1:**
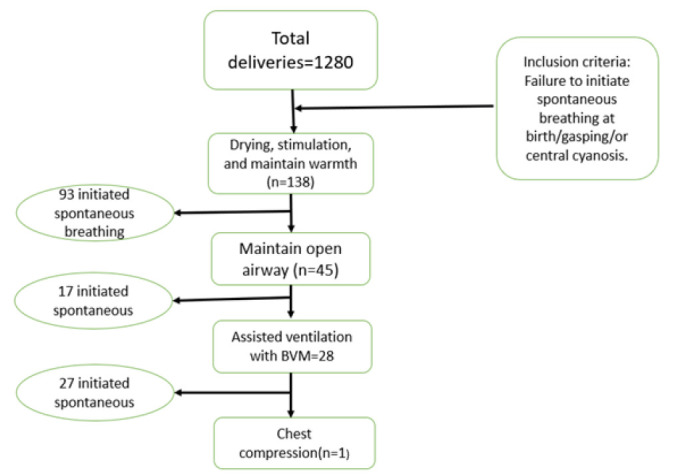
Flow Chart of Neonates Who Underwent Resuscitation

Nurses who were involved in neonatal resuscitation located in the labour ward and obstetric theatre consented to the study. All newborns who met the criteria for neonatal resuscitation based on the WHO guidelines (Figure 1), such as failure to initiate spontaneous breathing at birth/gasping, floppy, and/or central cyanosis. Babies who were born with anomalies incompatible with life and still births were excluded.^20^

Key to abbreviation: BVM, Bag Valve Mask.

### Sample Size Estimation

#### Number of Cases Observed

The minimum number of neonatal resuscitation cases to be observed was calculated using a Kish-Leslie formula. (21) A pilot study was done at MNH to ascertain the proportion of newborns requiring neonatal resuscitation to get a more representative sample size. A total of 557 deliveries were recorded in two months, with 47 newborns requiring neonatal resuscitation, making a proportion of 8.43%.



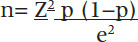



Whereby:

n=minimum sample size

z=standard normal deviation, which corresponds to 1.96 [at 95% CI]

*e*=marginal error assumed to be 5%

P Prevalence obtained from a pilot study

z= 1.96 p= 8.34% ε = 5%



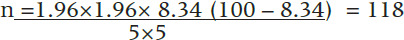



n = 118 newborns.

Assuming non-response is 15%, then the response rate, R, is 85%

N= 118 x 1/0.85 = 138

Total number of NR cases observed = 138

#### Number of Healthcare Providers

The same formula was applied, using a proportion from a study done in Dodoma, Tanzania, where the adequate knowledge on neonatal resuscitation among providers was 40.1%.(22) The estimated minimal sample size was 369, and calculating the same for a finite population was done as follows:



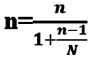



Where:

n = Adjusted minimal sample size for finite population

*n*= initial sample size for infinite population=369

N=Total population size=57



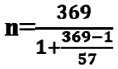



Number of providers recruited in the study (n) = 49

### Data Collection

All consenting study participants were provided with a paper-based standard questionnaire that captured information regarding demographics, such as age, sex, qualification, years of nursing experience, previous training in neonatal resuscitation, and the number of neonatal resuscitations performed.

The knowledge was assessed using standard questions derived from the HBB guidebook, 2nd Edition for neonatal resuscitation, focusing on the assessment of a newborn, which provides an approach for NR similar to the national guideline.^23^ The questions focused on the identification of a child requiring help, preparation of equipment, decision and action to be undertaken for those requiring help, initial steps of neonatal resuscitation, bag-mask ventilation, and chest compression. One mark was awarded for a correct response and zero for a wrong one. A minimum score of 75 % was regarded as adequate knowledge. This was derived after reviewing similar studies where a range score above 70–80% was used to determine an adequate level of knowledge.^24,25^ The principal investigator and a trained assistant provided the questionnaire to the participant after the completion of neonatal resuscitation.

The WHO standard checklist for assessing clinical knowledge and skills for basic newborn resuscitation, printed in a Clinical Practice Basic Newborn Resuscitation Guidebook, was adopted in the study.^26^ The checklist was used to evaluate neonatal resuscitation practice daily.

Health care providers (HCPs) who agreed to participate in the study were observed during neonatal resuscitation; the observation was non-intrusive, in which the participants were observed carrying out their usual routine practice without interference. To minimise the Hawthorne effect, the two research assistants were recruited among nurses working in the same institution to ensure participants’ familiarity. Additionally, the training of the assistants was done in the two study areas to allow the acclimatization of the study participants. Finally, the specific observed steps were not shared with the participants. The observation assessment focused on the preparation of appropriate equipment, keeping the baby warm, airway management, and BMV and chest compression. For the practice that was deemed harmful, the observer sought help from another provider. Two research assistants, one at the labour ward and another at the obstetric theatre, were used for data collection. The assistants were nurses with background training in NR, and they were trained on how to observe neonatal resuscitation against a predetermined checklist. To minimise inter-observer variability, all the research assistants were trained together and rated the same participants during the early phase of the study in the presence of the primary investigator (PI) and received objective feedback. The used checklist had clear instructions on the observed item, and the PI conducted periodic quality checks during data collection and offered feedback.

Two points were given to a step that was correctly done, one point if incorrectly done, and zero points if not done. The practice was regarded to be adequate as per guidelines if the provider scored at least 75% of the performed steps on the neonatal resuscitation checklist. The cut-off point score was determined based on previous studies.^25,27^

### Data Analysis

The questionnaires were coded and entered into IBM SPSS Statistics for Windows version 26.0 (IBM Corp, Armonk, NY, USA), which was used for analysis. The categorical variables were summarised using frequency distribution tables and statistical diagrams. The chi-square and Fisher's Exact tests were used to determine the association between the providers’ characteristics and practice. The logistic regression models were used for multivariable analysis. Providers’ characteristics with *p*-value < 0.2 on a bivariate analysis were subjected to a multivariable model. Using a 95% confidence interval, a *P* value of <.05 was considered statistically significant.

### Ethical Considerations

The MUHAS ethical research committee provided the ethical clearance (MUHAS-REC-07-2021-762) to conduct the study, and the Executive Director of MNH granted permission to conduct the study at MNH(MNH/TRCU/Perm/2021/103).

Participation in the study was entirely voluntary, and each individual gave a written informed consent to that effect. Participants were given a unique ID to ensure anonymity. Throughout the study period, confidentiality was maintained, and information gathered was kept in a computer with a password. Participants were free to leave the study at any time. To ensure the consenting process doesn't affect the practice, the specifics of the data collection process weren't disclosed to the participants.

## RESULTS

### Demographic Characteristics of Nurses Working in the Delivery Suites at Muhimbili National Hospital

The study included 49 participants who performed 138 cases of NR, of whom the majority were aged between 25 to 34 years, and were female. Based on their qualification, most were assistant nursing officers and nursing officers, equally represented. A little over half of the participants had a bachelor's degree in nursing, and most participants were working in the obstetric theatre. Most of the participants had more than five years of working experience within the delivery suite (Table 1).

**TABLE 1:** Demographic Characteristics of Nurses in the Delivery Suites

Variable	Frequency (n)	Percentage (%)
Age		
25–34	25	51.0
35–45	19	38.8
>45	4	10.2
Gender		
Male	24	49.0
Female	25	51.0
Designation		
ANO	18	36.7
NO	18	36.7
SANO	7	14.3
Intern nurse	6	12.2
Qualification		
Bachelor	25	51.0
Diploma	24	49.0
Working station		
Labour Ward	18	36.7
Theater	31	63.3
Work experience in delivery' suites		
1–2	10	28.6
3–5	15	30.6
>5	20	40.8

With regards to formal training on neonatal resuscitation, 41 (89.1%) had prior training, the majority were trained on HBB 35 (85.4%), most had trained once and 2–3 times 17 (41.5%), and 17 (41.5%) respectively, and the majority had their last training more than two years 31 (75.6%). The majority reported having attended in-house training on neonatal resuscitation 40 (81.6%), and most of them have reported either attending twice or once a year 11 (30.6%) and 11 (30.6%) respectively. The majority of the participants had performed more than 15 cases of neonatal resuscitation, 25 (51%). The majority of the participants were aware of the available guidelines for neonatal resuscitation 46 (93.9%)(Table 2).

**TABLE 2:** Training in Neonatal Resuscitation, the Number of Performed Resuscitations, and Awareness of Available Guidelines

Variable	Frequency (n)	Percentage (%)
Formal training in neonatal resuscitation		
Yes	41	89.1
No	8	10.9
Tvpes of training		
HBB	35	85.4
BEmONC	6	14.6
Number of neonatal resusdtation(s)		
1	17	41.5
2–3	17	41.5
>3	7	17.0
Last training		
6 months	3	7.3
1 yrs	7	17.1
2 yrs	31	75.6
In-house training		
Yes	40	81.6
No	9	18.4
Frequency of training		
Monthly	6	16.6
Quarterly	8	22.2
Six months	11	30.6
Yearly	11	30.6
Number of performed neonatal resuscitation		
0–5	5	10.2
5–10	12	24.5
10–15	7	14.3
>15	25	51.0
Awareness of availability of neonatal resuscitation guidelines		
Yes	46	93.9
No	2	4.3
I don't know	1	2.2

### The overall practice of neonatal resuscitation among nurses at MNH

**FIGURE 2:**
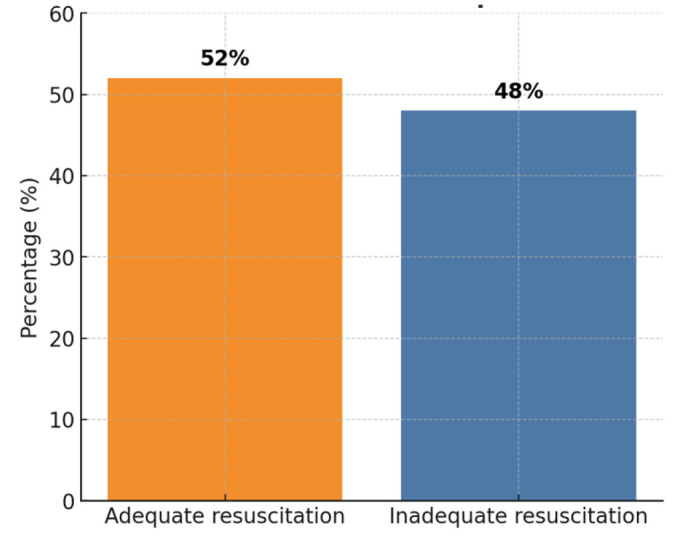
Overall Neonatal Resuscitation Practice

The practice was assessed in comparison with the standard practice. In all the cases, only 72 (52%) cases were adequately performed to meet the required standard (Figure 2).

### Equipment preparation before resuscitation

**FIGURE 3:**
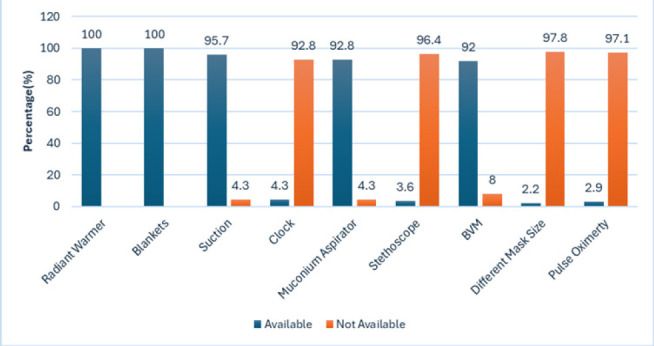
Availability of Essential Neonatal Resuscitation Equipment During Resuscitation

In the observed cases, warm blankets and radiant warmers were prepared in all cases. Suctions were prepared in 95.7% of cases, meconium aspirator in 96.4%, appropriate size BVM in 97.8%, and different size masks were prepared in 97.1% of cases. Equipment that was least prepared included a clock, which was only available in 7.2% of cases, a stethoscope in 8% of cases, and pulse oximetry in 7.2% of cases (Figure 3).

Key to abbreviation: BVM, Bag Valve Mask

### Observed neonatal resuscitation practice on individual steps

The study found that most practitioners called for help in 65.2% of cases, and only 6.5% started the clock. Most newborns were correctly dried, with 82.6% correctly dried, but the wet towel was discarded in only 38.4% of cases, and 99.3% were kept under a radiant warmer. Initial assessments were correct in 49.3% of cases; the heart rate was never assessed in the rest of the cases.

The newborns who didn't respond to stimulation (n = 45) proceeded to the subsequent steps. The study found that in 73.3% of newborns, the airway was opened correctly, 28 needed gentle suction, and all needed assisted ventilation. However, in 32.1% of cases, rescue breaths were given correctly, and reassessment was done correctly in 27.2% of cases. Only one newborn required chest compression, whose rate-to-ventilation ratio was incorrect, and documentation post-resuscitation was done in 2.9% of cases (Table 3).

**TABLE 3:** Neonatal Resuscitation Practice on Individual Steps Among Health Care Providers at Delivery Suites

Variable	Frequency (n)	Percentage (%)
Call for help		
Done	90	65.2
Not done	48	34.8
Start clock		
Done	9	6.5
Not done	129	93.5
Dry the baby		
Done	114	82.6
Not done	1	0.7
Incorrectly done	23	16.7
Discard the wet cloth		
Done	53	38.4
Not done	85	61.6
Wrap in a warm cloth		
Done	73	52.9
Not done	36	26.1
Incorrectly done	29	21
Put under warmer		
Done	137	99.3
Not done	1	0.7
Initial assessment		
Done	68	49.3
Not done	3	2.2
Incorrectly done	67	48.6
Neutral position		
Done	33	73.3
Not done	7	15.6
Incorrectly done	5	11.1
Ventilation within one minute		
Done	28	100
Gentle Suction		
Done	28	100
Correct Mask size		
Done	28	100
Good mask cover		
Done	26	93
Incorrectly done	2	7
Five rescue breaths		
Done	9	32.1
Incorrectly done	19	67.9
Observed chest rise		
Yes	28	100
No	0	0
Reassessment		
Done	6	27.2
Incorrectly done	22	72.8
Repeat rescue breath		
Done	3	27.2
Incorrectly done	8	72.8
Reassessment		
Done	3	27.2
Incorrectly done	8	72.8
CPR 3:1 ratio		
Incorrectly done	1	100
Reassessment		
Incorrectly done	1	100
Documentation		
Done	4	2.9
Not done	134	97.1

### Factors Associated with Neonatal Resuscitation Practice

The study showed that the factors which are statistically significantly associated with NR were practical working experience, level of knowledge on NR, formal training on NR, and in-house training on NR, (Table 4).

**TABLE 4:** Association Between Neonatal Resuscitation Practice and Health Care Providers' Characteristics

Variable	Neonatal Resuscitation		X2	*P value*
	Adequate	Inadequate		
Age				
25–34	34 (47.2%)	30 (45.5%)	0.06	.97
35–45	28 (38.9%)	27 (40.9%)		
>45	10 (13.9%)	9 (13.6%)		
Gender				
Male	30 (41.7%)	31 (47%)	0.393	.531
Female	42 (58.3%)	35 (53%)		
Provider level				
ANO	28 (38.9%)	26 (39.4%)	0.949	.814
NO	30 (41.7%)	26 (39.4%)		
SANO	12 (16.7%)	10 (15.2%)		
Intern nurse	2 (2.8%)	4 (6.1%)		
Qualification				
Diploma	39 (54.2%)	35 (54.5%)	0.003	.964
Degree	33 (45.8%)	30 (45.5%)		
Work Station				
Labour	31 (43.1%)	26 (39.4%)	0.19	.663
Theater	41 (56.9%)	40 (60.6%)		
Working experience				
1–2 years	11 (15.3%)	18 (27.3%)		
3–5 years	29 (40.3%)	12 (18.2%)	8.729	.013
>5 years	32 (44.4%)	36 (54.5%)		
Level of knowledge on NR				
High	48 (66.7%)	29 (43.9%)	7.212	.007
Low	24 (33.3%)	37 (56.1%)		
**Fisher's exact test Variable**	**Neonatal Resuscitation**		*P Value* (**2-sided**)	*P Value* (**1-sided**)
	**Adequate**	**Inadequate**		
Formal training on NR				
Yes	70 (97.2%)	57 (86.4%)	.026	.019
No	2 (2.8%)	9 (13.6%)		
In-house training on NR				
Yes	69 (95.8%)	57 (86.4%)	.069	.046

Key to abbreviation: NR, neonatal resuscitation.

In the univariate analysis, working experience 3 to 5 years (cOR 0.368; 95% CI, 0.161 to 0.839; *P*=.017) was associated with lower odds of inadequate practice, which was statistically significant. Additionally, having high knowledge on NR (cOR 0.392; 95% CI, 0.197 to 0.781; *P*=.008) and formal training on NR (cOR 0.181; 95% CI, 0.038 to 0.871; *P*=.033) were protective factors for inadequate practice. In Multivariate analysis, only working experience 3 to 5 years (AOR 0.352; 95% CI, 0.148 to 0.836; *P*=.018) was statistically significantly associated with low inadequate practice. While high knowledge of NR and formal training on NR were associated with reduced odds (AOR 0.534; *P*=.097 and AOR 0.164; *P*=.180) of inadequate practice, the association was not statistically significant (Table 5).

**TABLE 5:** A Multivariable Analysis of Neonatal Resuscitation and the Providers' Characteristics

Variable	Neonatal Resuscitation		95% Cl cOR	*P Value*	95%CIAOR	*P Value*
	Adequate	Inadequate				
Age						
25–34	34 (47.2%)	30 (45.5%)	0.98 (0.352–2.734)	.97	–	–
35–45	28 (38.9%)	27 (40.9%)	1.071 (0.377–3.044)	.897	–	–
>45	10 (13.9%)	9 (13.6%)	1		1	
Gender						
Male	30 (41.7%)	31 (47%)	1.24 (0.632–2.431)	.531	–	–
Female	42 (58.3%)	35 (53%)	1		1	
Provider level						
ANO	28 (38.9%)	26 (39.4%)	1.114 (0.412–3.013)	.831	–	–
NO	30 (41.7%)	26 (39.4%)	1.04 (0.386–2.799)	.938	–	–
sano	12 (16.7%)	10 (15.2%)	1		1	
Intern nurse	2 (2.8%)	4 (6.1%)	2.4 (0.361–15.9)	.365	–	–
Qualification						
Diploma	39 (54.2%)	35 (54.5%)	1.015 (0.519–1.985)	.964	–	–
Degree	33 (45.8%)	30 (45.5%)	1		1	
Work station						
Labour	31 (43.1%)	26 (39.4%)	0.86 (0.435–1.696)	.663	–	–
Theater	41 (56.9%)	40 (60.6%)	1		1	
Working experience						
1–2 years	11 (15.3%)	18 (27.3%)	1.455 (0.598–3.537)	.408	0.991 (0.371–2.65)	.986
3–5 years	29 (40.3%)	12 (18.2%)	0.368 (0.161–0.839)	.017	0.352 (0.148–0.38)	.018
>5 years	32 (44.4%)	36 (54.5%)	1		1	
Level of knowledge on NR						
High	48 (66.7%)	29 (43.9%)	0.392 (0.197–0.781)	.008	0.534 (0.255–1.12)	.097
Low	24 (33.3%)	37 (56.1%)	1		1	
Formal training on NR						
Yes	70 (97.2%)	57 (86.4%)	0.181 (0.038–0.871)	.033	0.164 (0.012–2.31	.18
No	2 (2.8%)	9 (13.6%)	1		1	
In-house training on NR						
Yes	69 (95.8%)	57 (86.4%)	0.275 (0.071–1.65)	.062	1.449 (0.115–18.31)	.774
No	3 (4.2%)	9 (13.6%)	1		–	

Key to abbreviation: NR, neonatal resuscitation.

## DISCUSSION

### Neonatal Resuscitation Practice

The study found that more than half of the cases were adequately performed, similar to a study conducted in Ghana.^28^ However, this is higher than a study in Kenya by Muli, where less than half of nurses had adequate practice.^29^ Much lower findings were reported by Makele et al. in Tanzania and Sintayehu *et al*. in Ethiopia, where only one-third of providers and 11.2% of participants successfully performed neonatal resuscitation, respectively.^30,31^ Our study and these other studies also show that poor neonatal resuscitation skills are a challenge among nurse providers in SSA, leading to some neonatal deaths.

The finding that health care providers demonstrate inadequate neonatal resuscitation practice in nearly half of the cases (48%) is concerning with serious clinical implications. Neonatal resuscitation is a time-sensitive intervention where delays and incorrect interventions in the first minute of life can lead to preventable neonatal deaths and hypoxic-ischemic encephalopathy, which is associated with long-term neurodevelopmental disability or death. This observed gap in practice implies that almost half of newborns requiring neonatal resuscitation may not receive effective care. Similar deficiencies have been reported in other sub-Saharan African countries, which reflects a regional challenge that requires deliberate interventions.^31–33^

The practice of individual steps of neonatal resuscitation indicated that 82.6% of all newborns were gently dried with a dry towel. This finding was nearly the same as the one seen by Shukuku et al in Kenya, which showed a slightly higher number of 88% of newborns adequately dried. However, the wet towel was discarded in only 38.4% of cases. This was much lower compared to the Shukuku *et al* study, in which more than two-thirds of newborns the wet towels discarded. The difference can be attributed to the knowledge and training between these two populations.^34^ The importance of keeping the baby warm during resuscitation by discarding wet towels might not be known among our HCPs.

Inappropriate stimulation techniques, such as vigorously rubbing and patting, were observed in some cases, which was slightly higher compared to the study by Shukuku et al in Kenya. This shows a significant number of newborns are exposed to inappropriate practices during resuscitation that can harm their health.^34^

For the newborns who did not respond to initial stimulation, the airway was opened by placing the newborn in a neutral position correctly in most of the cases, and all who required suction were done correctly. The findings a similar to the study by Shukuku *et al* in Kenya, which showed airway opening by putting the child in a neutral position was correctly done in similar percentage of cases. However, the suction was done correctly in only 40% of cases. This could be explained by the higher number of newborns requiring suctioning (n=123) in the Shukuku *et al* study compared to our study (n=28).^34^

In the newborns who failed to initiate breathing, assisted ventilation was commended within a golden minute in all cases. This indicated a better understanding of these practitioners in early ventilation within a golden minute. The good mask seal was achieved in the majority of cases, however, the ventilation breaths were correctly given in only one-third of cases.

In the study by Shikuku *et al*, bag and mask ventilation was initiated for all newborns who did not initiate breathing after airway opening; however, just more than half 54% were initiated within the golden minute. This was lower compared to that of our study, in which the researcher attributed this to the poor understanding of the initiation of ventilation with the golden minute among the study participants.^34^

Similarly, a study by Basu reported similar findings concerning the practice of assisted ventilation. The study revealed that most of the participants, 79.3% lack skills in positive pressure ventilation. Also, similar results were reported by Namuguzi *et al*, in which 72% of the participants did not ventilate at the required rate.^35,36^

Ventilation is one of the crucial steps in neonatal resuscitation and has a huge impact on neonatal outcomes. It provides oxygenation and ventilation to a newborn and helps the newborn to initiate spontaneous breathing. These studies indicated that this is one of the challenging steps among our providers; providers were either not ventilating at a required rate, ventilating without observing chest rise, or having too much ventilation volume.

Only a few newborns required further course of rescue breaths. This study revealed that most providers lack the skills to provide positive pressure ventilation, which is the most crucial step in neonatal resuscitation and will affect the outcome of the newborn with birth asphyxia.

Only one newborn required chest compression, which was incorrectly done because the provider failed to observe a 3:1 chest compression to ventilation ratio. A study by Suresh *et al* on the evaluation of knowledge and practice on neonatal resuscitation among nurses showed poor performance in the step of chest compression. Similar findings were reported by Basu in his study, in which most of the participants were poor at chest compression steps in neonatal resuscitation. Similar findings in the studies indicate that most nurses still lack skills in chest compressions in line with the standard guidelines.^35,37^

Documentation post-resuscitation was done in only 4 (2.9%) cases. This showed there was no documented information for the majority of the newborns who were resuscitated. The study by Bergulund *et al* on neonatal resuscitation assessment: documentation and early paging must improve! in Sweden, showed documentation was faulty in many cases; there was no documentation in 5.1% and 40% of the documentation was incomplete.^28^ The numbers in this study are much higher compared to our study, based on the difference in the study setting. The observed low findings in our study are attributed to the fact that in the observed delivery information chart, there was a section for documentation of APGAR score, but no section to record interventions done. This shows there is a problem concerning documentation after newborn resuscitation, which requires intervention through the development of a record tool that will capture this information

Another study by Avila-Alvarez et all a systematic review looking into documentation during neonatal resuscitation, showed incomplete documentation and missing important details that transpire during resuscitation. This lack of documentation impairs the continuity of care of the newborn when transferred from one point to another or transfer care is transferred from one HCP to another.^38,39^

### Factors Associated with Neonatal Resuscitation

The study showed that knowledge of neonatal resuscitation was statistically significantly associated with the neonatal resuscitation practice. This was reflected in the chi-square test and univariate analysis, which showed that high knowledge of neonatal resuscitation was protective against inadequate practice. Similar findings were reported in other studies by Muli and Sintayehu *et al*, respectively.^31,40^ This underpins the importance of having background theoretical knowledge for timely decision-making and interventions during emergencies. However, the lack of statistical significance in the multivariate analysis suggests that knowledge alone may not guarantee adequate practice; other factors, such as practical experience and regular exposure, may be equally important.

Formal Training on neonatal resuscitation was also found to be statistically significantly associated with neonatal resuscitation practice. The finding showed that formal training on neonatal resuscitation was associated with low odds of inadequate practice, which is consistent with other studies.^40,41^ The association was weakened after adjusting for other variables. This reflects the contribution of other factors that interplay to get a favourable result. Additionally, the finding underscores the need for regular refresher training to maintain competency. Similarly, in-house training showed a significant association with the practice, which signifies that even less formal workplace reinforcement skills can contribute positively.^42^

Notably, working experience of 3 to 5 years was associated with adequate practice of neonatal resuscitation and was the only significant predictor of adequate neonatal resuscitation on the multivariate analysis. The study showed that the providers with 3 to 5 years’ work experience were less likely to have inadequate practice. A similar relationship was found in a study by Joho et al and Mzurikwao et al. This reflects the role of ongoing exposure in developing competence.^43,44^

No significant association was found between providers’ demographic characteristics, such as age, gender, provider education level, qualification, or workstation, and neonatal resuscitation practice. Similar findings were reported in other studies.^(27,45)^ This suggests that targeted interventions should focus on training and skills reinforcement rather than providers’ demographics.

### Study Limitations

While this study as provided insight into the practice of NR, it has some limitations. First of all the study was conducted in a single centre, which is likely to limit the generalizability of the findings. Secondly, although the data were collected with the PI and two well-trained research assistants to ensure quality control, still there was a potential for observational bias. Thirdly, it was not possible to measure the long-term outcome of the resuscitation among affected newborns. Finally, as there is a chance of seasonal variation in the practice as the study was carried in a specific period that might limit the generalization of the findings.

## CONCLUSION

While a little over half of the nurses in delivery suites demonstrate adequate neonatal resuscitation practice, it is a matter of a concern that there considerable inadequate performance. Provided the time-sensitive nature of neonatal resuscitation and its role in preventing birth asphyxia, this observed gap poses a significant clinical implication, such that nearly half of newborns in need of resuscitation might be exposed to ineffective interventions. These findings call for deliberate targeted interventions to strengthen competence. Only working experience of 3 to 5 years was identified as an independent predictor of practice implying that continuous hands-on exposure remains crucial.

Based on the findings, we recommend structured refresher training programs, routine competency assessment among providers in delivery suited, on-the-job mentorship for those experienced providers guiding novice ones, and promoting in-house simulation sessions. Identification of local champions on neonatal resuscitation will ensure sustainability. Additionally, we recommend intervention studies that will look into the rate of decay of the neonatal resuscitation skills and the appropriate timeline for refresher training. Finally, we advocate for hospitals to embed neonatal resuscitation performance metrics in the ongoing quality improvement initiatives and health policies that institutionalize neonatal resuscitation as a mandatory requirement for continuous professional development for providers working in delivery suites.
